# Pollutants May Have Caused Morphological Abnormalities in Some Polychaete Species (Annelida) Collected from Cilacap, Central Java, Indonesia

**DOI:** 10.21315/tlsr2025.36.1.15

**Published:** 2025-03-30

**Authors:** Joko Pamungkas, Atang, Eko S. Wibowo, Misika Alam, Sri Lestari

**Affiliations:** 1 Research Centre for Biosystematics and Evolution, National Research and Innovation Agency. Jl. Raya Jakarta–Bogor Km.46, Cibinong, Bogor, West Java 16911, Indonesia; 2 Faculty of Biology, Jenderal Soedirman University. Jl. DR. Soeparno No. 63, Grendeng, Banyumas, Central Java 53122, Indonesia; 3 Academy of Health Analysts, An Nasher. Jl. Pondok Pesantren Tarbiyahtul Banin, Kaliwadas, Cirebon, West Java 45611, Indonesia

**Keywords:** Annelida, *Diopatra claparedii*, Marine Pollution, *Perinereis aibuhitensis*, Polychaeta

## Abstract

Morphological abnormalities in *Perinereis aibuhitensis* (Grube 1878) (Nereididae) and *Diopatra claparedii* Grube 1878 (Onuphidae) were observed in the specimens collected from the intertidal habitat around Donan Creek in Cilacap City, Central Java Province, Indonesia. The *P. aibuhitensis*, which is not supposed to have branchiae, possesses digitate branchiae on its dorsum, and lacks eyes. To our knowledge, the presence of branchiae in the genus *Perinereis* and the shape of the feature has never been reported anywhere else. Furthermore, the *D. claparedii*, which is supposed to have the most developed branchiae on its dorsal anterior region, lacks the feature. The species also lacks both prostomial and peristomial appendages, and has various anomalous cirri. While the abnormalities in the *P. aibuhitensis* are likely to be associated with the hypoxic condition of the animal’s habitat, the anomalies in the *D. claparedii* appear to be more related to the exposure to pollutants, particularly heavy metals. Taxonomic investigations are required to reveal the polychaete species richness in this area, and may identify species that have the potential to be used as biological indicators of coastal water pollution in southern Java.

**Highlights**

- Morphological abnormalities in two polychaete species were observed in the specimens collected from the intertidal habitat around Donan Creek in Cilacap City, Central Java Province, Indonesia.
- *Perinereis aibuhitensis* (Grube 1878), which is not supposed to have branchiae, possesses digitate branchiae on its dorsum, and lacks eyes.
- *Diopatra claparedii* Grube 1878 (Onuphidae), which is supposed to have most developed branchiae on its dorsal anterior region of the body, lacks the feature. The animal also lacks both prostomial and peristomial appendages, and has various anomalous cirri.

## INTRODUCTION

Donan Creek is part of the Segara Anakan Lagoon (SAL), i.e., a semi-closed estuary situated between Nusakambangan and Java Islands in Central Java Province. The creek—often regarded as a river by the locals despite the absence of a natural stream of freshwater—is the easternmost of the lagoon and is connected to Penyu Bay, which is part of the Indian Ocean. Alongside the creek lie mangrove forests as well as settlements, harbours, traditional markets, rice fields and factories. Donan Creek, like many other coastal waters in the country, constantly receives domestic, agricultural and industrial wastes from land. Over 50 contaminants, mostly polycyclic aromatic compounds (PACs) consisted mainly of polycylic aromatic hydrocarbons (PAHs), were reported to be present in the water, sediment and macrobenthic invertebrates inhabiting the SAL (Dsikowitzky *et al*. 2011). Moreover, various studies also revealed that the concentrations of particular heavy metals in the eastern part of the lagoon were above the threshold level (e.g., Hernawati 2014; Widiyanto 2014; Alam 2015; Syakti *et al*. 2015; Piranti *et al*. 2020).

Polychaete worms (Annelida) are a group of macrobenthic invertebrates inhabiting the SAL. The animals were abundantly found in the eastern part of the lagoon, particularly Donan Creek (Nordhaus *et al*. 2009; Pamungkas 2013). Most of the polychaete species in the SAL, however, remain unidentified due to limited taxonomic information on Indonesian species, which creates difficulty in identifying local species – see reviews of taxonomic studies on Indonesian polychaetes by Pamungkas and Glasby (2019) and Siallagan *et al*. (2023). Polychaete specimens obtained in Nordhaus *et al*. (2009) and Pamungkas (2013) were merely identified to both family and genus levels, and to date there are only two polychaete species of the SAL identified to the species level, i.e., *Polymastigos javaensis* Pamungkas 2015 (family Capitellidae), and *Diopatra claparedii* Grube 1878 (family Onuphidae; firstly reported to occur in Indonesian waters by Pamungkas *et al*. 2023).

During two different sampling occasions to Donan Creek, we came across two polychaete species with some morphological abnormalities belonging to the families Nereididae and Onuphidae. In this article, we aimed to describe both species and discussed possible factors causing the abnormalities.

## MATERIAL AND METHODS

**Figure 1:**
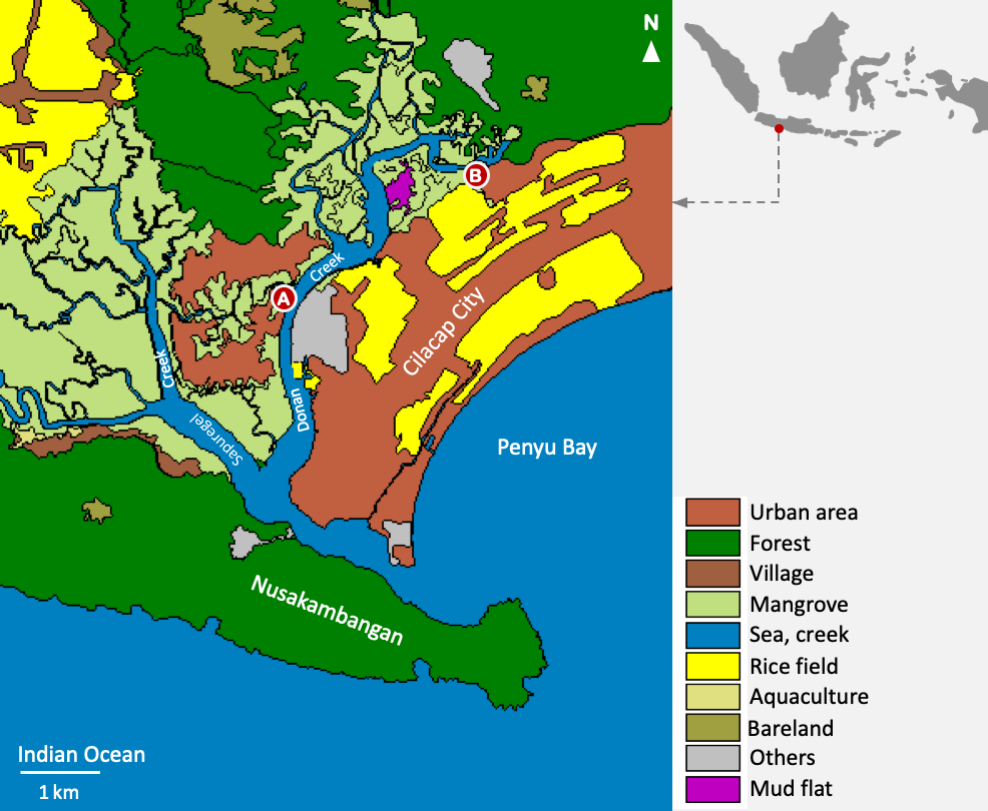
Map of the sampling sites (red circles). (A) Mangrove habitat across the oil refinery of Cilacap; (B) Mangrove habitat in Jeruklegi Village *Source*: Basic map was provided by Erwin R. Ardli.

A single nereidid specimen was collected across the oil refinery of Cilacap (7°41′32″S, 108°59′3″E) on 29 October 2013, whereas the onuphid specimen was obtained from Jeruklegi Village (7°39′53.5″S, 109°02′01.9″E) on 4 June 2021 (Fig. 1). Both worms were collected during a low tide from the intertidal mangrove habitat using a shovel, and were fixed using formalin 4% for approximately 24 h. The animals were then rinsed using tap water and transferred into vials with alcohol 70%. Afterwards, the specimens were examined under both stereo and compound microscopes using the identification keys of Fauchald (1977) and Hutchings *et al*. (1991) to identify the nereidid specimen, and Budaeva and Fauchald (2011) to identify the onuphid specimen. Both specimens are deposited at the Museum Zoologicum Bogoriense (MZB) in Cibinong, Bogor, West Java, Indonesia. However, the nereidid specimen went missing during the migration of the museum’s zoological collections to a new building (the morphological observation was completed before it went missing).

## SYSTEMATICS

Order Phyllodocida Dalles 1962

Family Nereididae Blainville 1818

Genus *Perinereis* Kinberg 1865

***Perinereis aibuhitensis*** **(Grube 1878)**

### Material examined

1 (SA-01), Cilacap City, Central Java Province, 7°41′32″S, 108°59′3″E, coll. Joko Pamungkas, 29 October 2013.

### Diagnosis

**Figure 2:**
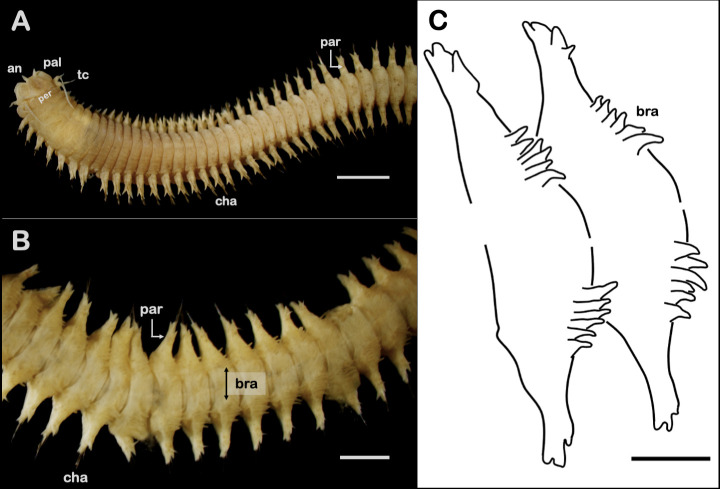
Anomalous *Perinereis aibuhitensis*. (A) Anterior part of the animal (dorsal view); (B) Dorsum of the middle part of the animal showing unusual branchiae (black arrow); (C) Close-up of branchiae. Scale bars: A = 2 mm, B = 1 mm, C = 0.5 mm. *Abbreviation*: an = antenna; bra = branchiae; cha = chaetae; pal = palp; par = parapodium; per = peristomium; tc = tentacular cirri;.

The nereidid species found in the present work shows morphological characteristics as follows. Body anterior part (almost complete), tapering posteriorly, 8 cm long with 131 chaetigers. Colour in alcohol pale yellow. Prostomium pear-shape with reduced eyes, 1 pair of antennae about ¼ of prostomium length, 1 pair of biarticulated palps, 4 pairs of tentacular cirri (3 short, 1 reaches chaetiger 3) (Fig. 2A). Except area VI, eversible pharynx with conical paragnaths on both rings arranged as follows: Areas (I) 2 relatively small paragnaths in a longitudinal line; (II) 9 and 11 paragnaths; (III) 13 paragnaths in 3 rows, with few additional ones arranged laterally, separated from central patch; (IV) 19 and 22 paragnaths; (V) 3 relatively bigger paragnaths in triangular; (VI) 2 biggest and very short bar paragnaths in a transverse row; (VII–VIII) 24 paragnaths in 2 rows.

Peristomium well-developed. Parapodia biramous with well-developed lobes and pointed dorsal and ventral cirri. Chaetae in anterior and posterior chaetigers identical with homogomph spiniger in notopodia and homogomph spiniger, heterogomph spiniger and heterogomph falciger in neuropodia. Simple digitate branchiae start from chaetiger 41, appear from dorso-lateral region of body. Length of branchiae increase posteriorly; number of branchiae in one fascicle increase posteriorly from about 3 in chaetiger 41, to about 6 or 7 in more posterior chaetigers (Figs. 2B and 2C). Pygidium broken.

### Habitat

The intertidal mangrove habitat with sandy mud substrate across the oil refinery of Cilacap City.

### Local distribution

The species was commonly found around Donan and Sapuregel Creeks in Cilacap City, Central Java Province, yet it was misidentified as *Neanthes* sp. in Nordhaus *et al*. (2009) and Pamungkas (2013) because the shape of the paragnaths on area VI looks like cones rather than bars. Horst (1924) documented the occurrences of this species in Jakarta (old name: ‘Batavia’) and Makassar (South Sulawesi Province).

### Remarks

**Figure 3:**
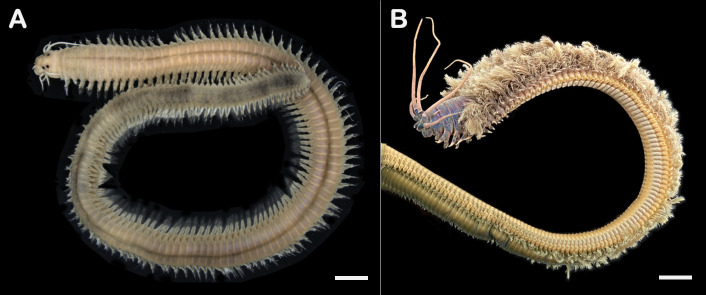
Local species with normal morphologies. (A) *Perinereis aibuhitensis*; (B) *Diopatra claparedii*. Scale bars: A = 1 mm, B = 5 mm.

The most striking features of the nereidid specimen in the present study that show the abnormality of the animal are the presence of simple digitate branchiae arising from the dorso-lateral region of the body segments, and the reduced eyes. Members of the genus *Perinereis* do not normally possess branchiae (Bakken & Wilson 2005; Fig. 3A). Two genera of the family Nereididae whose members have branchiae are *Dendronereis* and *Dendronereides*, but the shape of the branchiae is normally pectinate, i.e., having narrow parallel projections resembling teeth of a comb (Fauchald 1977). Thus, to our best knowledge, the presence of simple digitate branchiae in *Perinereis*, and the shape of the feature itself is firstly reported in the present study.

The nereidid specimen in the present work was almost mistakenly identified as *Neanthes* sp. due to the shape of paragnaths in area VI being very short and almost identical to cones (see Hutchings *et al*. 1991). The paragnath arrangement of the present specimen, nonetheless, agrees well with that of *P. aibuhintensis*. Specimens collected from the same area identified as *Neanthes* sp. in Pamungkas (2013) are most likely *P. aibuhitensis*, and have been reported to be the most common nereidid species in the eastern part of the SAL. Despite further sampling efforts conducted in the same area between July and August 2023, we did not find any other anomalous *P. aibuhitensis*, suggesting that the anomalous species tends to be due to abnormalities, and is unlikely to be a new genus.

Order Eunicida

Family Onuphidae Kinberg 1865

Genus *Diopatra* Audouin & Milne Edwards 1833

***Diopatra claparedii*** **Grube 1878**

### Material examined

1 (MZB. Pol. 00239), Jeruklegi Village, Cilacap City, Central Java Province, 7°39′53.5″S, 109°02′01.9″E, coll. Eko S. Wibowo, 4 June 2021.

### Diagnosis

**Figure 4:**
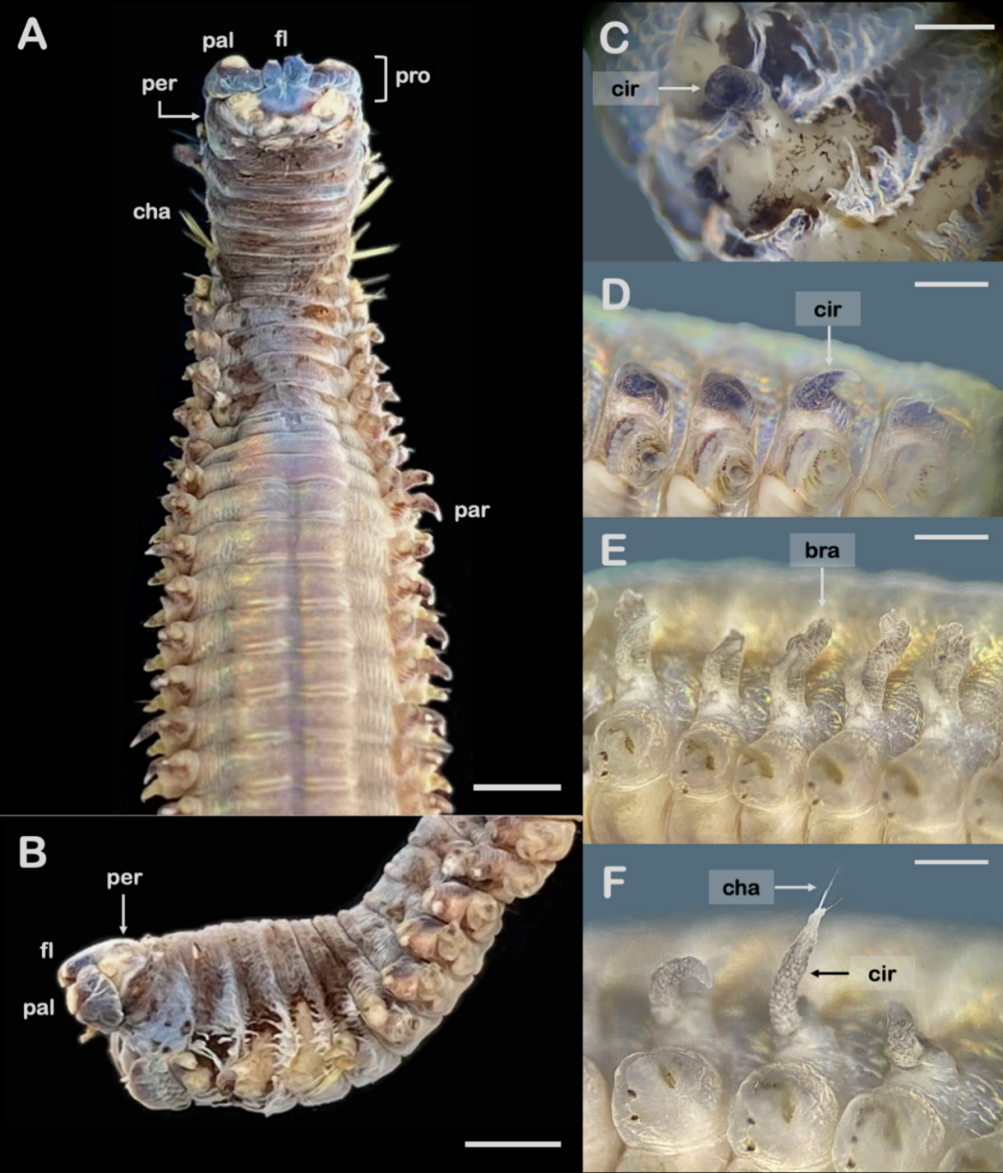
Body parts of anomalous *Diopatra claparedii*. (A) Anterior dorsal view showing no branchiae up to chaetiger 37; (B) Anterior lateral view showing no branchiae; (C) finger-like cirrus; (D) Cirri; (E) Branchiae; (F) Chaetae on the tip of a cirrus. Scale bars: A & B = 2.5 mm; C, D, E = 1 mm; F = 0.5 mm. *Abbreviation*: cha = chaetae; cir = cirrus; fl = frontal lips; pal = palp; par = parapodium; per = peristomium; pro = prostomium.

Tube conical, elongated, tapering posteriorly. Anterior part of tube tough, thick, leathery and reinforced with fibres and bits of mangrove leaves; the posterior part thin and papery. A few mangrove leaves present near tube opening. Specimen incomplete (anterior part) with about 170 chaetigers, 14.8 cm long and 0.9 cm wide at the widest area (i.e., around chaetiger 85). Dorsum of prostomium purplish; dorsum of peristomium to chaetiger 9 purplish brown; other parts of body generally pale yellow. Prostomium anteriorly rectangular with two paired subulate frontal lips (right lip about twice bigger than left one), two paired undeveloped palps and (probably) removed antennae. Peristomium without appendages (Fig. 4A). First four anterior parapodia (chaetiger 1–4) look broken or less developed (Fig. 4B), followed posteriorly with a few parapodia with lip-like prechaetal lobes and subulate post chaetal lobes. Dorsal cirri of chaetiger 1–12 show various abnormalities, i.e., either undeveloped (or probably broken), fingerlike (Fig. 4C) or oval sphere. Chaetiger 13 posterior wards with subulate dorsal cirri (Fig. 4D) despite some random abnormalities, becoming shorter posteriorly (like a small mound). Ventral cirri absent (either not developed or probably broken). Relatively small spiralled branchiae starting from chaetiger 38 posterior wards in various positions (Fig. 4E), i.e., present randomly on either left or right side only, or both sides, or absent; branchiae with 2 whorls only. Chaetiger 1–9 with slender simple chaetae present in various positions, i.e., on upper side only, on both upper and lower sides, and on basal of undeveloped (or broken) dorsal cirri. Smaller, slender, simple chaetae also present randomly at tip of some dorsal cirri (Fig. 4F). Limbate chaetae and pectinate chaetae with funnel-like combs present. Falcate and bidentate hooks present.

### Habitat

The intertidal mangrove habitat with muddy substrate. Bigger specimens of the species inhabit the estuary and can only be sampled using hands (see how an experienced local caught the worms at https://www.youtube.com/watch?v=1cBQx1yDOqU&t=2s).

### Local distribution

The species was found around Donan and Sapuregel Creeks in Cilacap City (pers. obs.).

### Remarks

The most obvious anomalous appearance of the *D. claparedii* in the present study is the absence of branchiae along the dorsum of the body’s anterior part; the feature is generally supposed to be the most developed in that body region (see Fig. 3B). The animal lacks most of prostomial and peristomial appendages, i.e., antennae, palps and peristomial cirri, yet this may be because the features break off as scars are seen on their respective positions (thus unrelated to the abnormality of the species). Moreover, the right frontal lip of the animal is more swollen than the left one, and the dorsal cirri are in various malformations, i.e., undeveloped (or probably broken), fingerlike and oval sphere. Another anomalous feature includes the presence of slender simple chaetae in a very unusual location, i.e., at the tip of some dorsal cirri. The animal is identified as *D. claparedii* by the presence of small spiralled branchiae and distinct pectinate chaetae with funnel-like combs, which are unique to the species (Paxton 2002; Idris & Arshad 2013; Pamungkas *et al*. 2023). The animal was found along with *D. claparedii* specimens collected during the study of Wibowo *et al*. (2022). The species was originally identified as *Diopatra* sp. by the authors, and was later identified as the first record of *D. claparedii* from Indonesia by Pamungkas *et al*. (2023).

## DISCUSSION

The occurrence of polychaete species with morphological abnormalities has been widely reported across the globe. These anomalous animals belong to various families such as Amphinomidae (e.g., Méndez *et al*. 2009), Capitellidae (e.g., Reish *et al*. 1974), Eunicidae (e.g., Mohammad 1981), Nereididae (e.g., Mohammad 1981; Baoling *et al*. 1985; Creaser & Bean 1990; Oliveira 2009; Pamungkas 2011; Sawestri 2013; Coutinho & Santos 2014), Oenonidae, Opheliidae, Serpulidae (e.g., Mohammad 1981) and Onuphidae (e.g., Paxton 2002; Pamungkas *et al*. 2023). Nereididae, apparently, has become the most frequently reported polychaete family with anomalous species. This is, in part, because Nereididae is one of polychaete families with the most species (Pamungkas *et al*. 2019). Also, certain nereidid species were reported to be resistant to pollutants (Dean 2008), a factor that is often considered responsible for morphological abnormalities in polychaetes (e.g., Baoling *et al*. 1985; Sawestri 2013; Coutinho & Santos 2014).

*Perinereis aibuhitensis* collected in the present work has reduced eyes. This is in line with the findings of some other studies suggesting that morphological abnormalities in nereidids may occur in various body parts such as prostomium and peristomium (e.g., antennae, eyes, tentacular cirri), parapodia and chaetae. The abnormalities are typically in the form of reduction, addition and/or anomalous shape and size of body features (e.g., Mohammad 1981; Baoling *et al*. 1985; Creaser & Bean 1990; Pamungkas 2011; Sawestri 2013; Coutinho & Santos 2014). The presence of digitate branchiae, as well as their occurrence in *P. aibuhitensis*, has nonetheless never been reported in any previous taxonomic investigations, and seems to be due to ecophenotypic factors, i.e., environmental conditions affecting the phenotype of organisms. Since branchiae are normally used as a respiratory extension, the occurrence of this feature in *P. aibuhitensis* may be related to the low oxygen level in the animal’s habitat. The average level of oxygen in the eastern part of the SAL, including Donan and Sapuregel Creeks as well as the connecting channel to the Indian Ocean, was about 3 mg/L (Piranti *et al*. 2020). While this number is generally lower than the oxygen level standard for marine life set by the Indonesian government, i.e., > 5 mg/L (Ministry of Environment 2004), the pore water of the substrate in which the *P. aibuhitensis* lived is estimated to be hypoxic. This, as reported by Pamungkas (2013), was indicated by the presence of some dominant polychaete species accustomed to living in a hypoxic environment, i.e., *Leitoscoloplos* sp. (Orbiniidae), *Paraprionospio* sp. (Spionidae) and *Polymastigos javaensis* Pamungkas 2015 (Capitellidae) – these species occurred exactly at the same site as the *P. aibuhitensis*.

Furthermore, morphological abnormalities in *D. claparedii* were probably first documented by Paxton (2002). In one paralectotype of the species, she observed that the left parapodium 2 has two postchaetal lobes, and the right parapodium 2 has two dorsal cirri. Pamungkas *et al*. (2023) recently discovered another anomaly. Several *D. claparedii* specimens collected from Donan Creek, Cilacap, were found to have no maxillae, making the maxillary formula becomes as follow: Maxilla I = 0–1 + 0–1, Maxilla II = 0–8 + 0–8, Maxilla III = 0–8 + 0, Maxilla IV = 0–8 + 0–8, Maxilla V = 0–1 + 0–1.

The *D. claparedii* in the present study shows more morphological abnormalities than those reported by Paxton (2002). While some abnormalities might be due to injuries by predators (e.g., the missing antennae and first few parapodia), some other ones seem to be related to malformations of the organs resulting from environmental pressure (e.g., the anomalous shape of dorsal cirri with random presence of slender simple chaetae at the tip of the cirri). We here also consider the absence of branchiae from chaetigers 1 to 37, as well as the random presence of relatively small branchiae from chaetiger 38 posterior wards, a malformation due to environmental pressure. In fact, many studies over decades demonstrated that polychaetes with morphological abnormalities often occurred in polluted areas (e.g., Mohammad 1981; Baoling *et al*. 1985; Creaser & Bean 1990; Oliveira 2009; Sawestri 2013; Coutinho & Santos 2014). Exposure to particular heavy metals such as copper, mercury and zinc was moreover known to be responsible for morphological abnormalities in various polychaete species (e.g., Reish *et al*. 1974; Geracitano *et al*. 2004; Méndez *et al*. 2009). A number of studies indicated that the concentrations of several heavy metals in the eastern part of the SAL, including cadmium, chromium, cuprum, iron, lead and mercury, were above the threshold level (e.g., Hernawati 2014; Widiyanto 2014; Alam 2015; Syakti *et al*. 2015; Piranti *et al*. 2020). Similarly, the levels of polychlorinated biphenyls, tributyltin, as well as nitrate and phosphate, were also reported to exceed the threshold (Piranti *et al*. 2020). Most of these substances are carcinogenic, and are likely to be the primary cause of the abnormalities in *D. claparedii*.

## CONCLUSION

We found two polychaete species collected from Donan Creek, Cilacap City, with firstly-reported types of morphological abnormalities. Whereas the abnormalities in *P. aibuhitensis* (especially the presence of digitate branchiae) are likely to be associated with the hypoxic condition of the animal’s habitat, the anomalies in *D. claparedii* (especially the malformations of some features) appear to be more related to exposure to pollutants, particularly heavy metals. Both *P. aibuhitensis* and *D. claparedii* are common species in the eastern part of the SAL, and have potential as bioindicator of the ecosystem’s health. As most polychaete species of the studied area remain unidentified, taxonomic investigations are required, and may discover other anomalous species and individuals in that industrial region, particularly because the anthropogenic activities around the creek remain relatively the same to date.
